# Antisense Oligonucleotide-Based Therapy on miR-181a-5p Alleviates Cartilage Degradation of Temporomandibular Joint Osteoarthritis *via* Promoting SIRT1

**DOI:** 10.3389/fphar.2022.898334

**Published:** 2022-06-15

**Authors:** Hexu Qi, Zhenxing Zhao, Lin Xu, Yue Zhang, Yifei Li, Li Xiao, Yu Li, Zhihe Zhao, Jie Fang

**Affiliations:** ^1^ State Key Laboratory of Oral Diseases, National Clinical Research Center for Oral Diseases, West China Hospital of Stomatology, Sichuan University, Chengdu, China; ^2^ College of Stomatology, Chongqing Medical University, Chongqing Key Laboratory of Oral Diseases and Biomedical Sciences, Chongqing, China; ^3^ Department of Pediatrics, Ministry of Education Key Laboratory of Women and Children’s Diseases and Birth Defects, West China Second University Hospital, Sichuan University, Chengdu, China; ^4^ Department of Stomatology, Sichuan Academy of Medical Sciences, Sichuan Provincial People’s Hospital, University of Electronic Science and Technology of China, Chengdu, China

**Keywords:** TMJOA, miR-181a-5p, antisense oligonucleotide, SIRT1, gene therapy

## Abstract

Temporomandibular joint osteoarthritis (TMJOA) condylar cartilage degeneration and abnormal subchondral bone pathological remodeling induce pain and joint dysfunction, and cartilage degeneration is considered irreversible. Very few therapeutic approaches are administrated in practice. Nucleotides have demonstrated considerable potential as a next-generation medication, and they have been applied in several models of osteoarthritis. There is a need to establish an effective protocol for TMJOA gene therapy. In the current study unilateral anterior crossbite (UAC) surgery was used to simulate mechanical stress-induced TMJOA in mice. Degeneration of condylar cartilage and destruction of subchondral bone were observed in damaged joints, and miR-181a-5p was elevated in chondrocytes. Intra-articular injection of miR-181a-5p antisense oligonucleotide (ASO) could reduce the cartilage damage and alleviate UAC-induced TMJOA progression, but it did not restore injured subchondral bone. Mechanically, miR-181a-5p evidently targeted the 3’ untranslated region of *Sirt1* directly, resulting in inhibition of silent information regulator 1 expression and promoting apoptosis by elevating p53-dependent signaling, indicating that miR181a-5p ASO promoted chondrocyte survival. The present study suggests that ASO-based gene therapy may be an effective TMJOA treatment.

## Introduction

Temporomandibular joint osteoarthritis (TMJOA) is considered one of the most serious temporomandibular joint (TMJ) disorders, and is characterized by progressive and degenerative changes in joints (Liu et al., 2021). The primary manifestations of TMJOA include condylar cartilage degeneration and abnormal subchondral bone pathological remodeling, which induce pain and joint dysfunction (Shi et al., 2017; Li et al., 2021). This joint dysfunction can significantly reduce quality of life, and degenerative changes in cartilage can reportedly be irreversible. To date only symptomatic therapies have been administrated in practice. It is important to develop and validate approaches to attenuate or alleviate the degradation of cartilage (Wang et al., 2015). Multiple risk factors have been implicated in the initiation and progression of TMJOA, including aging, sex, hormone levels, diet, immune activity, and excessive mechanical stress loading on joints (Chantaracherd et al., 2015). It has previously been reported that abnormal occlusal relationships contributed greatly to TMJ cartilage degradation, and that unilateral anterior crossbite (UAC) can induce chondrocyte apoptosis and osteoarthritis-like lesions in temporomandibular joint (TMJ) cartilage (Liu et al., 2021). A mouse model of TMJOA can be generated with minimal trauma via a simple operation, and a UAC mouse model can be similarly established, which serves as an animal model for the *in vivo* study of biological responses associated with excessive mechanical stress (Wang et al., 2014; Yang et al., 2020).

MicroRNAs (miRNAs) are an evolutionarily conserved group of small non-coding RNAs, and research investigating them has accumulated rapidly in recent years (Mestdagh et al., 2011). Several miRNAs evidently serve critical functions in the regulation of the chondrocyte mechanotransduction pathway. Disturbances of such miRNAs are demonstrably involved in the occurrence and development of osteoarthritis (Kopańska et al., 2017; Cazzanelli and Wuertz-Kozak, 2020). miR-181a-5p, a member of the miR-181 family, is reportedly highly expressed in injured lumbar facet joint and knee osteoarthritis cartilage in human and experimental animal samples (Nakamura et al., 2016; Nakamura et al., 2019). These findings imply that miR-181a-5p is a potential therapeutic target in patients with osteoarthritis. Previous studies indicate that antisense oligonucleotide (ASO) can inhibit gene expression by inducing enzyme-dependent degradation of targeted RNA *in vivo*, prompting speculation about its use in gene therapy (Kole et al., 2012; Bajan and Hutvagner, 2020; Dhuri et al., 2020). Nakamura et al. (Nakamura et al., 2019) reported that locked nucleic acid miR-181a-5p ASOs exhibit cartilage-protective effects in facet joint and knee osteoarthritis.

Despite the above-described observations, to date there is no reported gene therapy study on TMJOA. The current study investigated whether miR-181a-5p ASO could attenuate the progression of TMJOA induced by excessive mechanical stress, and attempted to identify downstream pathways and molecular mechanisms involving miR-181a-5p and TMJOA.

## Methods

### Mice

Forty 8-week-old C57/BL6 female mice weighing 18–22 g were randomly allocated to a UAC group, a SHAM group, a SHAM-SCO group, a UAC-SCO group, or a UAC-ASO group. All procedures were approved by the Ethics Committee of Sichuan University, China, and performed in accordance with institutional guidelines. UAC operations involving the placement of a metal tube bound to the left-side mandibular incisors to create an experimental anterior crossbite relationship among the left side incisors (Zhang et al., 2019) in the UAC, UAC-SCO, and UAC-ASO groups were all performed by the same animal surgeon. Identical surgical procedures were performed on mice in the SHAM and SHAM-SCO groups, without metal tube fixation.

### ASO Administration

In the UAC-SCO and SHAM-SCO groups 7.5 nmol/joint miR-181a-5p SCO was locally injected bilaterally into the TMJ area on the 4th week after UAC or sham surgery, to generate blank controls. In the UAC-ASO group the same amount of miR-181a-5p ASO was injected via the same procedure. All SCO and ASO were administered twice a week for 6 weeks. SCO and ASO were synthesized by RIBOPharm Corp., China. The respective sequences of SCO and ASO were ACT​CAC​CGA​CAG​CGT​TGA​ATG and TAA​CAC​GTC​TAT​ACG​CCC​A.

### Dual Luciferase Reporter Assays

SIRT1 wild-type and mutant 3′ untranslated regions (3′UTRs) were constructed after the corresponding sequences containing the forecasted or mutant binding sites were inserted into pEZX-FR02 vector (GeneCopoeia Co., Ltd.). HEK293T cells were seeded into 24-well plates at a confluence of approximately 70%, then co-transfected with luciferase reporter and mmu-miR-181a-5p mimics or NC mimics (RiboBio, China) using Lipofectamine 3,000. After incubation for 48 h, luciferase activities were measured using the Dual-Luciferase Reporter Assay System (Vazyme Biotech, China) and a microplate reader.

### Cell Culture

Primary mandibular condylar chondrocytes were isolated from condyles cartilages of Sprague Dawley rats (Chantaracherd et al., 2015; Wang et al., 2015; Shi et al., 2017; Li et al., 2021; Liu et al., 2021). After being harvested from the mandibular, cartilage pieces were digested with trypsin for 30 min and 0.2% collagenase II for 1.5 h. The digestion was stopped by adding complete media containing 10% fetal bovine serum (FBS). Following centrifugation, chondrocytes were maintained under standard cell culture conditions at 37°C and 5% CO2 in 10%FBS/ Dulbecco’s modified Eagle’s medium and used from passage 1 to passage 4.

### Treatments of Cells in the *in Vitro* Experiments

The chondrocytes were divided five groups: negative control (NC) siRNA (RiboBio), Sirt1 siRNA (RiboBio), NC mimic (RiboBio), rno-miR-181a-5p mimic (RiboBio), rno-miR-181a-5p mimic + SRT 1720 (MedChemExpress, HY-15145). Chondrocytes were fused to 50–60%, then transfected with siRNA or mimic using Lipofectamine RNA iMAX transfection reagent (Invitrogen) individually. In the final group, the cells were treated with miR-181a-5p mimic, and additionally, SRT 1720 was added at a dose of 5 μM per 10^6^ cells. 72 h post transfection, cells were harvested to check Sirt1 and P53 expression levels by western blot and qPCR, combined with detecting cell apoptosis by TUNEL staining.

### Tissue Preparation and Histological Staining

Bilateral temporomandibular joint tissues in each group were removed and fixed with 4% paraformaldehyde solution for 12 h, then decalcified in decalcifying solution (50.0% sodium citrate, 20.5% formic acid, and 29.5% ddH_2_O) for 4 weeks. During the decalcification process the decalcifying solution was exchanged every second day. The specimens were dehydrated via an alcohol gradient series, embedded in paraffin, and continuous sections of 4.0 μm were cut on the coronal plane. Slices of the median area of the TMJ were selected and subjected to staining procedures.

Tissue sections were dewaxed with xylene, and rehydrated with gradient ethanol and water. Morphological changes in the TMJ were investigated via hematoxylin–eosin (HE) staining (G1120HE, Solarbio, China), and cartilage matrix was evaluated via Toluidine Blue (G3668, Solarbio, China) and Safranin-O/Fast Green (GG1371-5, Solarbio, China). After routine dehydration and sealing, a digital slide scanner (Pannoramic MIDI, 3D Histech) was used to acquire images of the sections.

### Immunohistochemistry

Immunohistochemistry analyses were performed to investigate the presence of silent information regulator 1 (SIRT1) and P53-positive cells within cartilage. After dewaxing, rehydration, and permeabilization in 1% Triton X-100, paraffin sections were treated with 3% H_2_O_2_ to block endogenous peroxidases, then blocked with 3% bovine serum albumin. The samples were then incubated with primary antibodies overnight at 4°C, then with biotinylated anti-rabbit immunoglobulin for 30 min at room temperature. The antibodies used were SIRT1 (1:200 dilution, 13161-1-AP, Protein Tech, China) and P53 (1:200 dilution, 10442-1-AP, Protein Tech, China). After treatment with peroxidase-labeled streptavidin, DAB and hematoxylin were added for color development. After routine dehydration and sealing, sections were imaged *via* a digital slide scanner (Pannoramic MIDI, 3D Histech).

### TUNEL Staining

To assess levels of condylar chondrocyte apoptosis, TUNEL staining of paraffin sections was performed in accordance with the instructions supplied with the Colorimetric TUNEL Apoptosis Assay Kit (Beyotime, C1098). Stained sections were then observed *via* a digital slide scanner (Pannoramic MIDI, 3D Histech). For *in vitro* experiments, TUNEL staining was used according to the instructions (One Step TUNEL Apoptosis Assay Kit, Beyotime, C1090) to assess the cell apoptosis. TUNEL-positive cells were observed and measured under the confocal microscope.

### Quantitative Real-Time PCR

Condylar cartilage was removed from joints with the aid of dissection microscopy. Total RNA was then extracted from it using the RNeasy Mini Kit (Qiagen, 74,104), and reverse transcribed by the SuperScript III First-Strand Synthesis System (Invitrogen, 18080051). MiR-181a-5p expression was determined using the All-in-One™ miRNA qRT-PCR Detection Kit (Genecopfollows) with the U6 internal control. The miR-181a-5p-specific forward primer sequence used was AAC​ATT​CAA​CGC​TGT​CGG​TGA​GT, and the universal reverse primer was provided in the kit.

For the *in vitro* experiments, RNA was extracted from chondrocytes using NucleoZOL (MNG, 740,404.200) and reverse transcribed by PrimeScript™ RT reagent Kit with gDNA Eraser (Perfect Real Time) (Takara, RR047A). Quantitative real-time PCR (qRT–PCR) was performed using TB Green®Premix Ex Taq™ II (Tli RNaseH Plus) (Takara, RR820). The sequences of primers used are the following:

Sirt1-F: 5′-AGA​GTT​GCC​ACC​AAC​ACC​TC-3′,

Sirt1-R: 5′-CAA​CAG​CCT​TGA​AAT​CTG​GGT-3’; P53-F:5′-GTCACCTCCACACCTCCACCTG-3′,

P53-R: 5′-GTC​ACC​TCC​ACA​CCT​CCA​CCT​G -3’; 18s-F:5′-TTGACGGAAGGGCACCACCAG -3′,

18s-R: 5′- GCA​CCA​CCA​CCC​ACG​GAA​TCG-3’.

### Western Blot Analysis

Cells were lysed with SDS Lysis Buffer (Beyotime, P0013G) containing protease inhibitors and sheared by sonication. The concentration of protein was determined using Enhanced BCA Protein Assay Kit (Beyotime, P0010) and a microplate reader. Western blot analysis was performed using the Wes Simple Western system (Protein Simple). All the reagents used are provided in the kit (DM-001, DM-002, SM-W004). The primary antibody used in the study included SIRT1 (1:20 dilution, 13161-1-AP, Protein Tech) and P53 (1:10 dilution, 10442-1-AP, Protein Tech) and GAPDH (1:50 dilution, 60004-1-Ig, Proteintech), and the secondary antibody was ab6789, ab6721 (Abcam). Results of Wes Simple Western System were obtained using the “Lane” function of the Compass software.

### Micro-Computed Tomography Analysis

After fixation with 4% paraformaldehyde solution for 48 h, specimens were stripped of soft tissue, leaving the TMJ condylar head structures intact. They were then scanned via micro-computed tomography (Skyscan1176, Bruker, USA), with source voltage 50 kV, source current 450 μA, AI 0.5-mm filter, pixel size 9 μm, and rotation step 0.400 degrees. The images obtained were reconstructed with NRecon software (Bruker microCT, Kontich, Belgium). Two cubic regions of interest (0.25 mm^3^) at the mid-points of the center and posterior condyle were selected, in accordance with previous studies (Zhang et al., 2013). Parameters including percent bone volume (BV/TV), bone surface/volume ratio (BS/TV), trabecular number (Tb.N), trabecular thickness (Tb.Th), and bone mineral density (BMD) were analyzed via CTAn software (Bruker microCT, Kontich, Belgium).

### Statistical Analysis

GraphPad Prism 9.0.0 (GraphPad Software, Inc.) was used for statistical analysis and data plotting. Statistical analyses were performed using Student’s t-test for comparisons between two groups, and one-way analysis of variance with Tukey’s multiple comparisons test for comparisons between ≥3 groups. Data are presented as means ± the standard deviation. *p* < 0.05 was considered statistically significant.

## Results

### Expression of MiR-181a-5p Increased in Condylar Cartilage of TMJOA Mice

**FIGURE 1:**
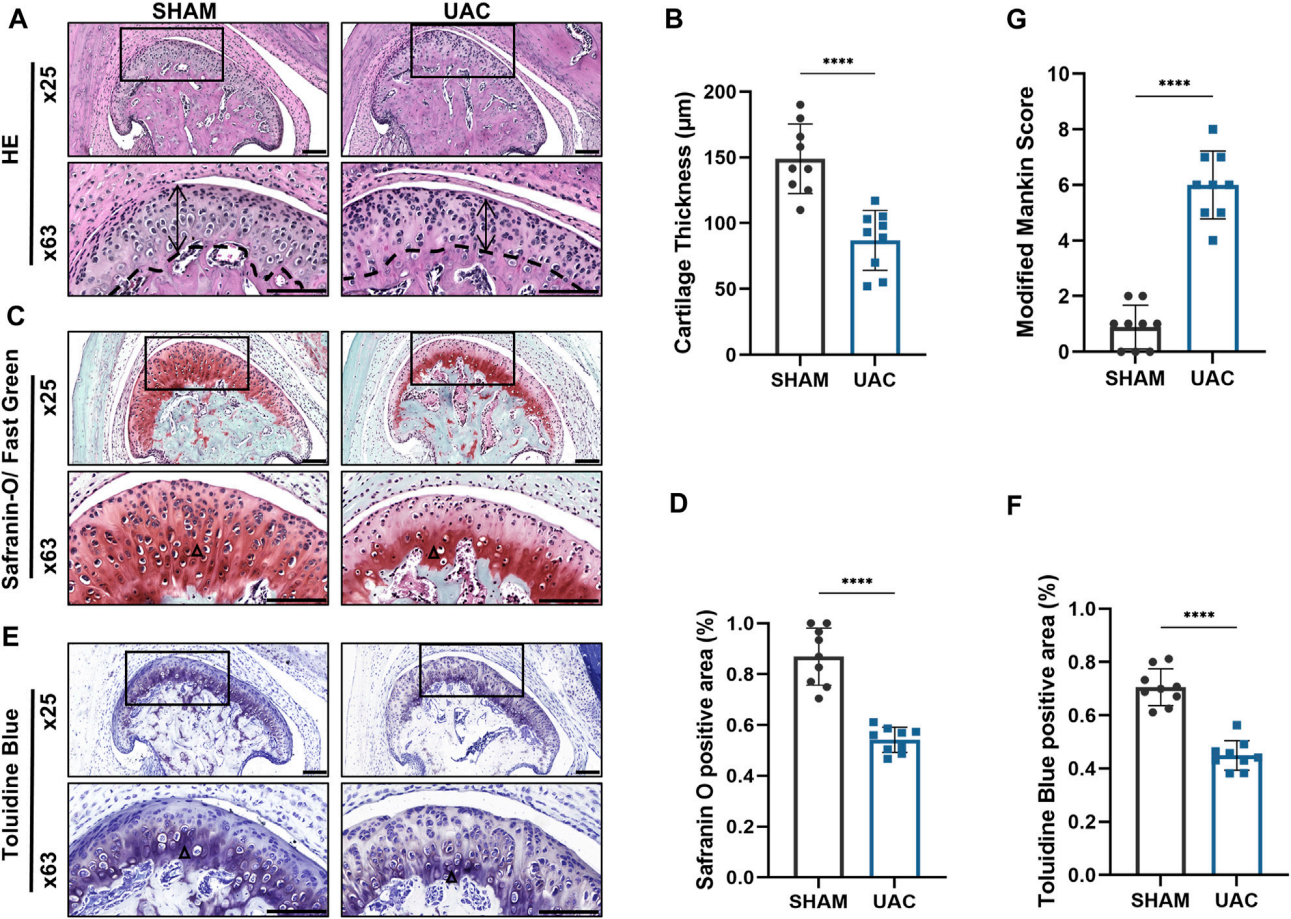
Morphological changes in the mouse condylar cartilage after the unilateral anterior crossbite (UAC) operation. **(A,C,E)** Representative sections with HE, Safranin-O/ Fast Green and Toluidine Blue staining of UAC and SHAM mice. Scale bar = 100 μm. For visual comparison, condylar heads in **(A,C,E)** are adjusted to the same orientation. Double-head arrow indicates the cartilage thickness; black triangle represents staining positive area. **(B)** Comparison of average cartilage thickness (μm) between UAC and SHAM groups. *n* = 9/ group. **(G)** The modified Mankin scores of the cartilage in two groups. *n* = 9/ group. **(D,F)** The proportion of the safranin O and Toluidine Blue positive area to the entire cartilage layer in UAC and SHAM groups. *n* = 9/ group. **(B,G,D,F)** Data presented as Scatter dot plot with bar (mean ± SD). Significance was determined using t-tests. *p < 0.05; **p < 0.01; ***p < 0.001; ****p < 0.0001.

**FIGURE 2:**
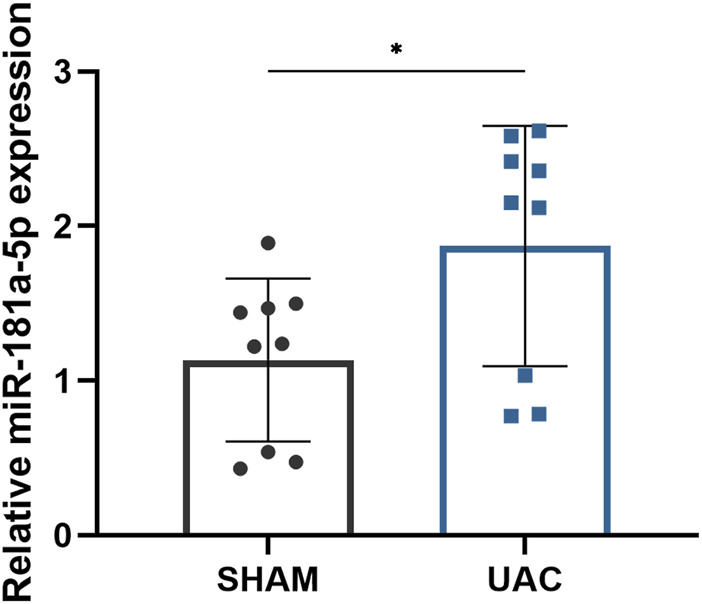
Comparison of relative miR-181a-5p expression in healthy and osteoarthritis (OA) condylar cartilage. Relative miR-181a-5p expression in healthy and OA condylar cartilage was determined by qRT-PCR using U6 as internal control. *n* = 9/ group. Data presented as Scatter dot plot with bar (mean ± SD). Significance was determined using t-tests. *p < 0.05.

HE staining revealed that the average width of the condylar cartilage was significantly thinner in the UAC group than in the SHAM group (*p* < 0.0001), combined with disorderly arrangement of chondrocytes, particularly an increased number of proliferating cells and aggregated clusters thereof, and cell-free areas (Figures 1A,B). Safranin-O and Toluidine Blue staining revealed a significant decrease in cartilage area in the UAC group compared to the SHAM group (*p* < 0.0001), which represented the degradation of proteoglycan in cartilage matrix (Figures 1C–F). The UAC group exhibited higher modified Mankin histological scores than the SHAM group (*p* < 0.0001) (Figure 1G). These results were consistent with typical features of osteoarthritis, and indicated the successful establishment of a TMJOA model *in vivo*. QRT-PCR data revealed the potential involvement of miR-181a-5p in TMJOA (Figure 2). miR-181a-5p expression was significantly greater in TMJOA cartilage tissue in the UAC group than in the SHAM group.

### MiR-181a-5p Binds Directly to the 3′UTR of SIRT1 and Inhibits Its Expression

**FIGURE 3:**
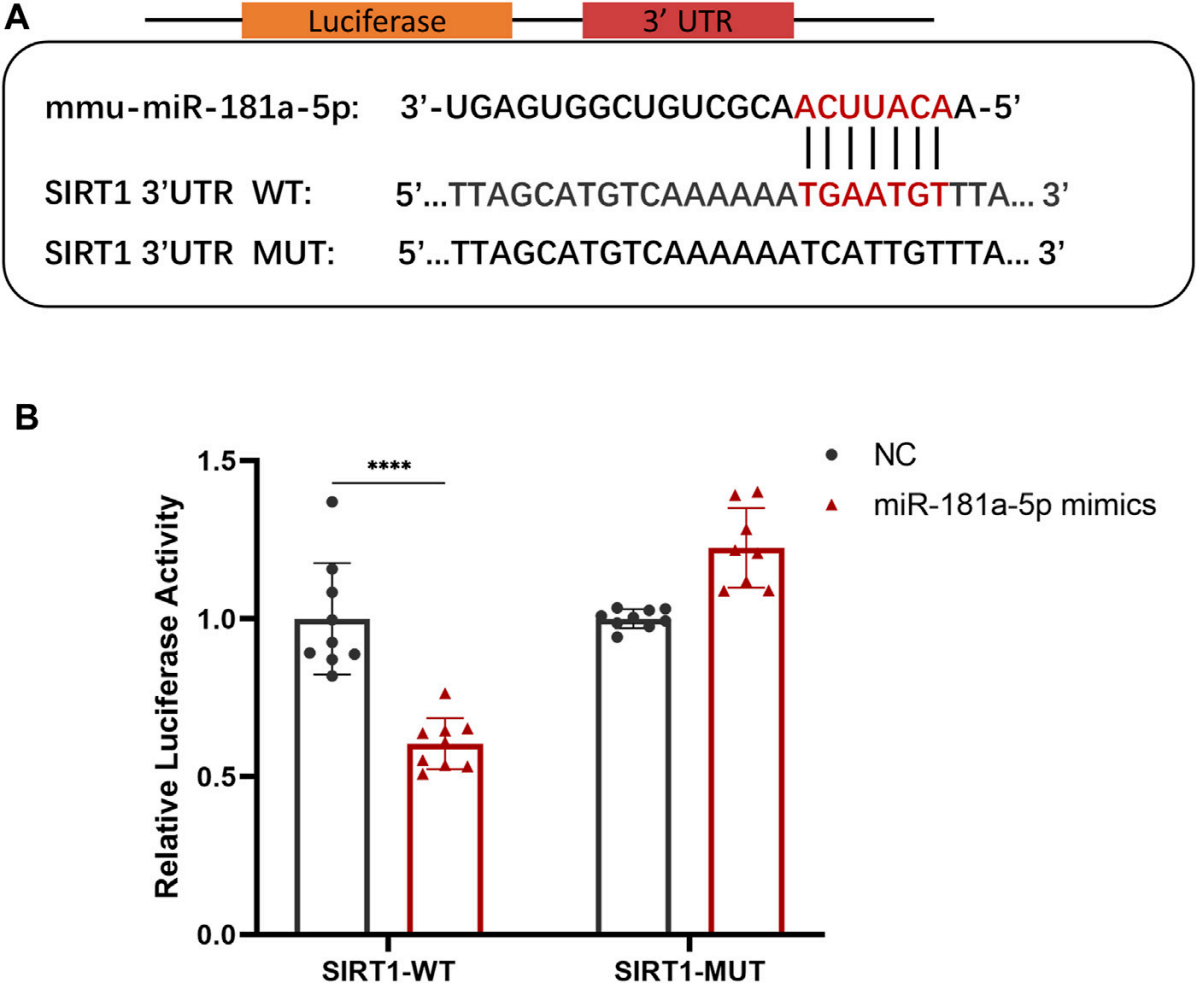
MiR-181a-5p Binds Directly to the 3′-UTR of SIRT1 and Inhibits its Expression. **(A)** Schematic representation of miR-181a-5p binding site in the SIRT1 3′UTR, which were predicted by database. **(B)** Relative luciferase activity detected using dual-luciferase reporter gene activity assay system. *n* = 9/group. Data presented as Scatter dot plot with bar (mean ± SD). Statistical analysis was performed using two-way ANOVA with a Tukey’s multiple comparisons test. ****p < 0.0001.

To explore the potential downstream mechanisms of miR-181a-5p, we used TargetScan (http://www.targetscan.org) and miRDB (http://www.mirdb.org/) databases to predict the target genes of miR-181a-5p and search for potential binding sites. There was a miR-181a-5p binding site in the SIRT1 3′UTR. On this basis, wild-type and mutant SIRT1 reporter plasmids were constructed for use in luciferase reporter assays (Figure 3A). Co-expression of miR-181a-5p and wild-type SIRT1 reporter plasmid significantly reduced relative luciferase activity (*p* < 0.0001), whereas co-expression of miR-181a-5p with the mutant SIRT1 plasmid did not differ significantly (Figure 3B). Together these results indicate that miR-181a-5p targets SIRT1 directly, and negatively regulates SIRT1 expression.

### MiR-181a-5p Stimulates Apoptosis of Chondrocytes by Targeting the SIRT1/P53 Pathway *In Vitro*

**FIGURE 4:**
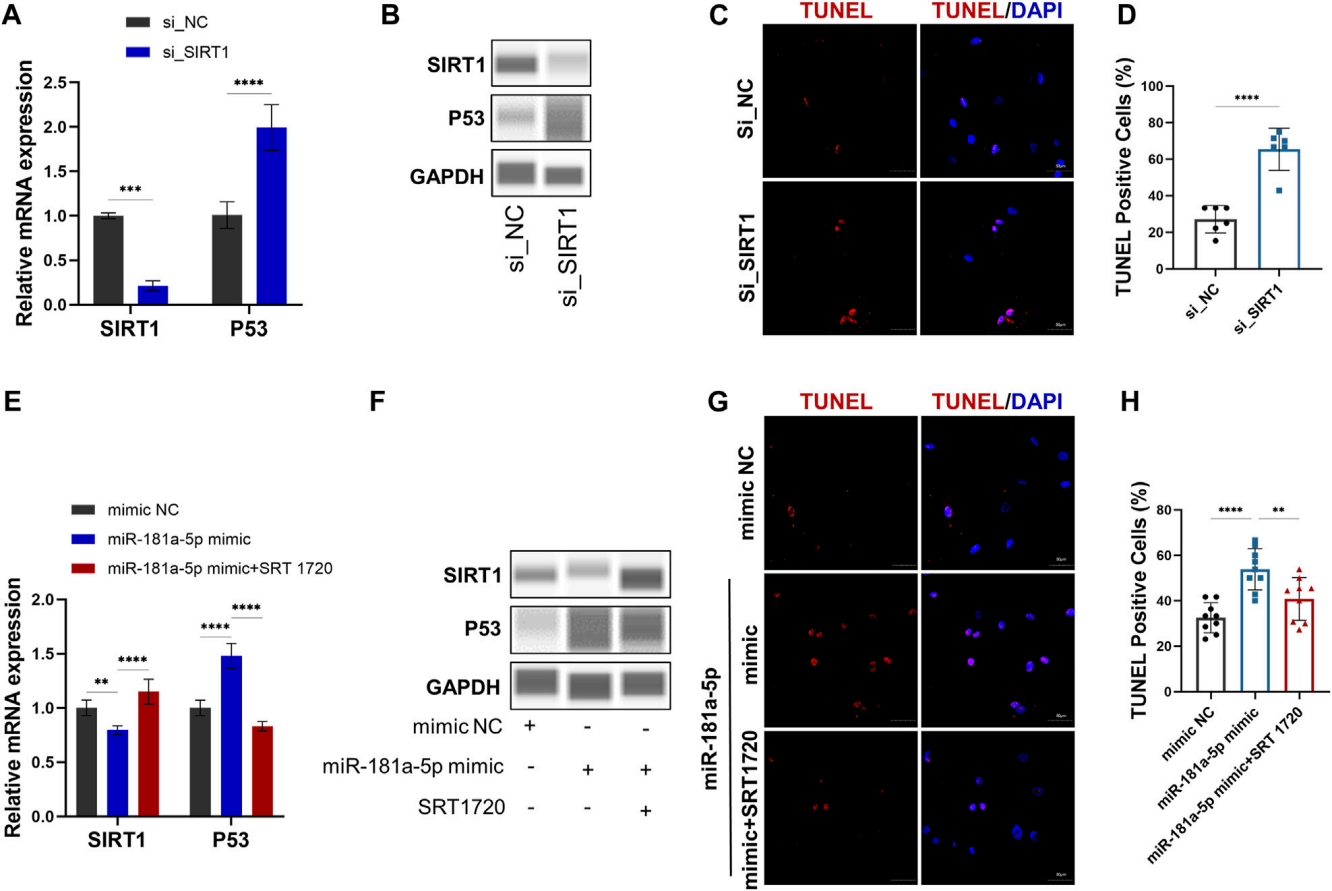
MiR-181a-5p stimulates apoptosis of chondrocytes by targeting the SIRT1/P53 pathway *in vitro*. **(A,E)** Relative mRNA expression of SIRT1 and P53 in chondrocytes. **(B, F)** Protein expression results of Wes Simple Western System using the “Lane” function of the Compass software. **(C,G)** Representative images of TUNEL staining in chondrocytes. (red, TUNEL-positive staining; blue, DAPI). Scale bar = 50 μm. **(D,H)** TUNEL-positive cell counts (%). **(D)** *n* = 6/ group. **(H)** *n* = 9/ group. Data presented as Scatter dot plot with bar (mean ± SD). T-test was employed for comparisons between two groups, one-way analysis of variance with Tukeys post test for multiple comparisons was used for groups of three. **p < 0.01; ***p < 0.001; ****p < 0.0001.

QRT-PCR (Figure 4A) and Western blot (Figure 4B) results demonstrate siRNA-mediated knockdown of SIRT1. Meanwhile, P53 mRNA (*p* < 0.0001) and protein expression significantly increased by SIRT1 knockdown (Figures 4A,B). TUNEL^+^ percentages were analyzed through TUNEL staining, which revealed a higher number (*p* < 0.0001) of apoptotic cells in the group treated with siRNA SIRT1 (Figures 4C,D). The results indicated that SIRT1/P53 pathway serves a regulatory role in chondrocyte apoptosis.

To further confirm the relation between miR-181a-5p and SIRT1, chondrocytes were transfected with the miR-181a-5p mimic or NC. Consistent with the luciferase reporter assays, the qRT-PCR (Figure 4E) and WB (Figure 4F) confirmed that SIRT1 expression was downregulated (*p* < 0.01) by miR-181a-5p mimic transfection. As in the knockdown of SIRT1, miR-181a-5p mimic transfection also resulted in upregulated P53 mRNA (*p* < 0.0001), protein level and TUNEL^+^ chondrocytes (*p* < 0.0001). However, these alterations were reversed by treatment with SRT1720, a selective SIRT1 agonist (Figures 4E–H). Taken together, miR-181a-5p promotes chondrocyte apoptosis through SIRT1/P53 pathway.

### MiR-181a-5p ASO Administration Alleviated Cartilage Degradation in UAC-Induced TMJOA Mice

**FIGURE 5:**
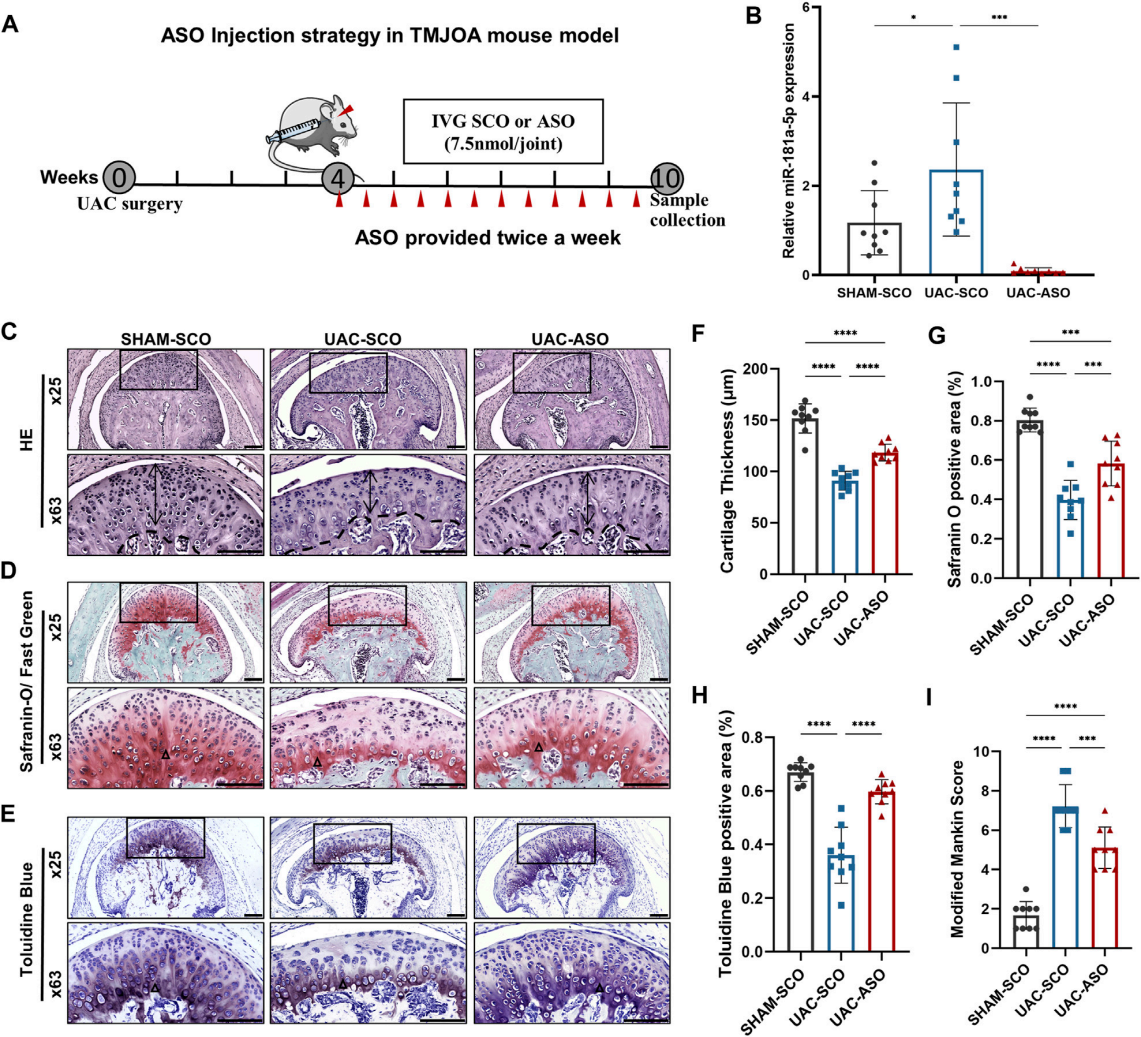
MiR-181a-5p ASO local injection alleviated cartilage destruction in UAC-induced TMJOA mice. **(A)** Schematic of intra-articular injection. Four weeks after UAC, ASO or SCO was given in TMJ cavity twice per week for 6 weeks and samples harvested at 10 weeks. Red triangles represent and injection sites and time points. **(B)** Relative miR-181a-5p expression in SHAM-SCO, UAC-SCO and UAC-ASO condylar cartilage by qRT-PCR using U6 as internal control. *n* = 9/ group. **(C–E)** Representative images showing HE, Safranin-O/ Fast Green and Toluidine Blue staining morphological change of the TMJ in SHAM-SCO, UAC-SCO and UAC-ASO mice. Scale bar: 100 μm. For visual comparison, condylar heads in **(C–E)** are adjusted to the same orientation. Double-head arrow indicates the cartilage thickness; black triangle represents staining positive area. **(F)** Comparison of average cartilage thickness (μm) in three study groups. *n* = 9/ group. **(G,H)** The percentages of the safranin O and Toluidine Blue positive area to the entire cartilage layer in SHAM-SCO, UAC-SCO and UAC-ASO groups were calculated. *n* = 9/ group. **(I)** The modified Mankin scores of the severity of TMJOA lesion in three study groups. *n* = 9/ group. **(F–I)** Data presented as Scatter dot plot with bar (mean ± SD). Statistical analysis was performed using one-way ANOVA with a Tukey’s multiple comparisons test. *p < 0.05; **p < 0.01; ***p < 0.001; ****p < 0.0001.

According to previous research, TMJOA manifestations can be observed 3 weeks post-UAC surgery in mice (Li et al., 2020). Therefore, we started bilateral miR-181a-5p ASO injections in the TMJ cavity at the 4th week after UAC, and they were administered twice a week for 6 consecutive weeks. A detailed representation of the injection schedule is shown in Figure 5A. qPCR indicated that relative expression of miR-181a-5p was significantly greater in condylar cartilage in the UAC-SCO group than in the SHAM-SCO group. Intra-articular injection of miR-181a-5p ASO attenuated the expression of miR-181a-5p (*p* < 0.001) in the UAC-ASO group (Figure 5B).

Based on this result, HE, Toluidine Blue, and Safranin-O/Fast Green staining were applied to TMJ sections in these three groups to evaluate associated pathological changes. UAC surgery induced significant TMJOA lesions, and visible local fissure-like defects. Surprisingly, a non-negligible change in TMJOA mice was evident after ASO intra-articular injection. The cartilage layer in UAC-ASO mice was thicker than that in UAC-SCO mice (*p* < 0.0001), and the number of chondrocytes in each layer recovered to an extent (Figures 5C,F). Safranin-O staining revealed a larger area of proteoglycan (*p* < 0.0001) compared to UAC-SCO mice (Figures 5D,G), and similar results were observed after Toluidine Blue staining (*p* < 0.0001, Figures 5E,H), indicating inhibition of cartilage matrix damage due to miR-181a-5p administration. There was a significant reduction in Mankin histological scores in the UAC-ASO group (*p* < 0.001, Figure 5I). In general, inhibition of miR-181a-5p expression by ASO alleviated the severity of condylar cartilage degeneration.

### MiR-181a-5p ASO Alleviates Chondrocytes Apoptosis in TMJOA by Targeting the SIRT1/P53 Pathway

**FIGURE 6:**
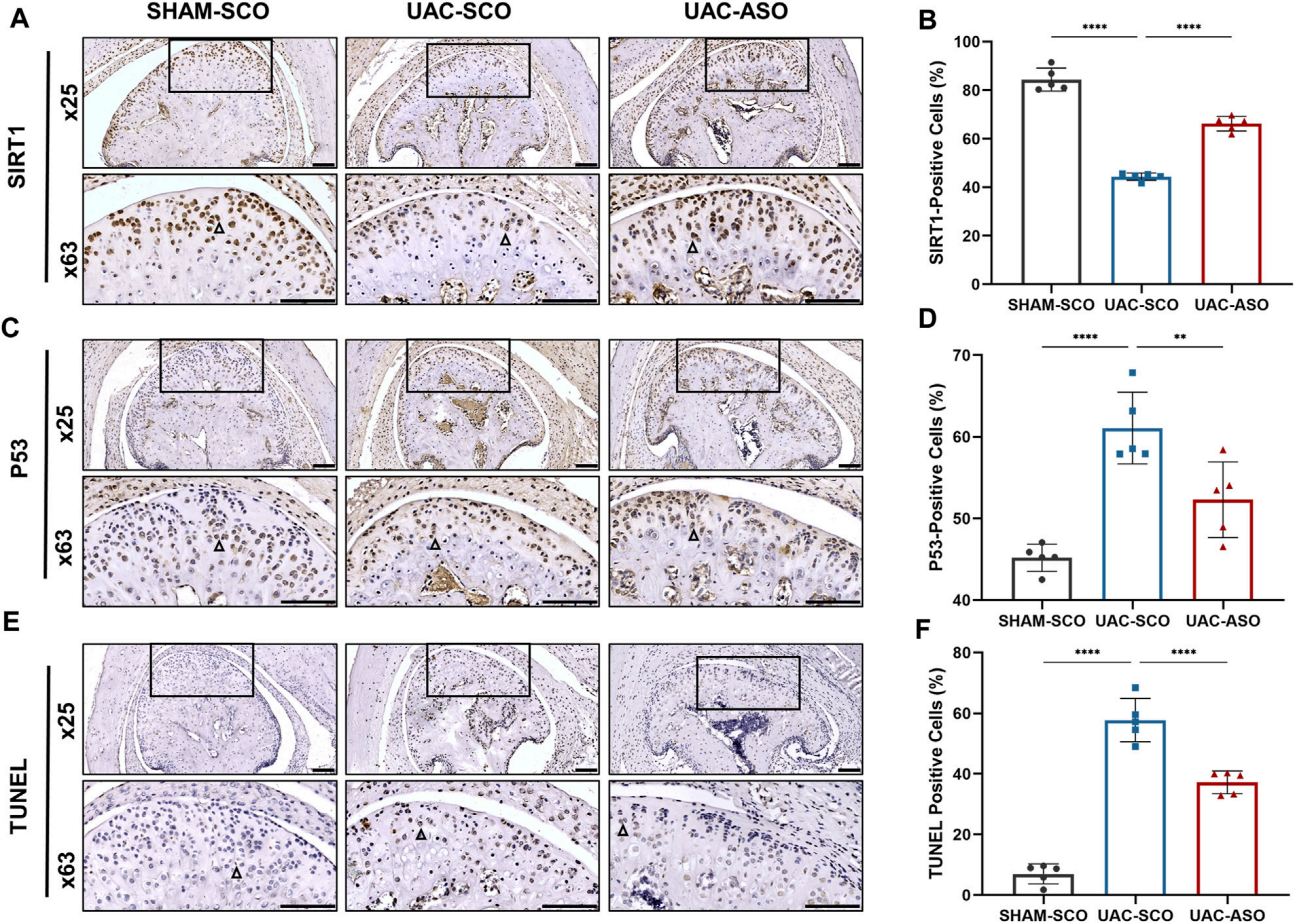
MiR-181a-5p affects chondrocyte apoptosis by targeting the SIRT1/ P53 signaling pathway in mice. **(A)** Representative images of SIRT1 immunohistochemistry (IHC) and **(B)** the percentage of SIRT1-positive IHC staining cells in the SHAM-SCO, UAC-SCO and UAC-ASO mice. Scale bar = 100 μm n = 5/group. **(C)** Representative images of P53 IHC and **(D)** the percentage of P53-positive cells in the in three study groups. Scale bar = 100 μm *n* = 5/group. **(E)** Representative images of TUNEL staining and **(F)** the percentage of TUNEL-positive cells in the in three study groups. Scale bar = 100 μm *n* = 5/group. For visual comparison, condylar heads in **(A, C, E)** are adjusted to the same orientation. Examples of IHC-positive cells are indicated by black triangles. **(B,D,F)** Data presented as Scatter dot plot with bar (mean ± SD). Statistical analysis was performed using one-way ANOVA with a Tukey’s multiple comparisons test. **p < 0.01; ***p < 0.001; ****p < 0.0001.

Immunohistochemical analysis of SIRT1 in TMJ sections revealed a reduction in the ratio of SIRT1^+^ chondrocytes in the UAC-SCO group compared to the SHAM-SCO group (*p* < 0.0001). Consistent with our *in vitro* results, these results indicating that activated expression of miR-181a-5p inhibited expression of SIRT1 in chondrocytes in the TMJOA model. The ratio of SIRT1^+^ chondrocytes was promoted in UAC mice after miR-181a-5p ASO administration (*p* < 0.0001, Figures 6A,B), which was also confirmed by dual luciferase reporter gene assays.

SIRT1 participated in cellular survival by inhibiting P53 and alleviating apoptosis under pathological stress. Immunohistochemical staining was performed to assess P53 expression, and TUNEL staining was performed to assess chondrocyte apoptosis. UAC induced elevated expression of P53 and associated chondrocyte apoptosis. Injection of miR-181a-5p ASO attenuated activation of the P53 pathway (*p* < 0.0001, Figures 6C,D) and associated chondrocyte apoptosis (*p* < 0.01, Figures 6E,F).

### MiR-181a-5p ASO Injection Restored Condylar Subchondral Bone Quality in TMJOA

**FIGURE 7:**
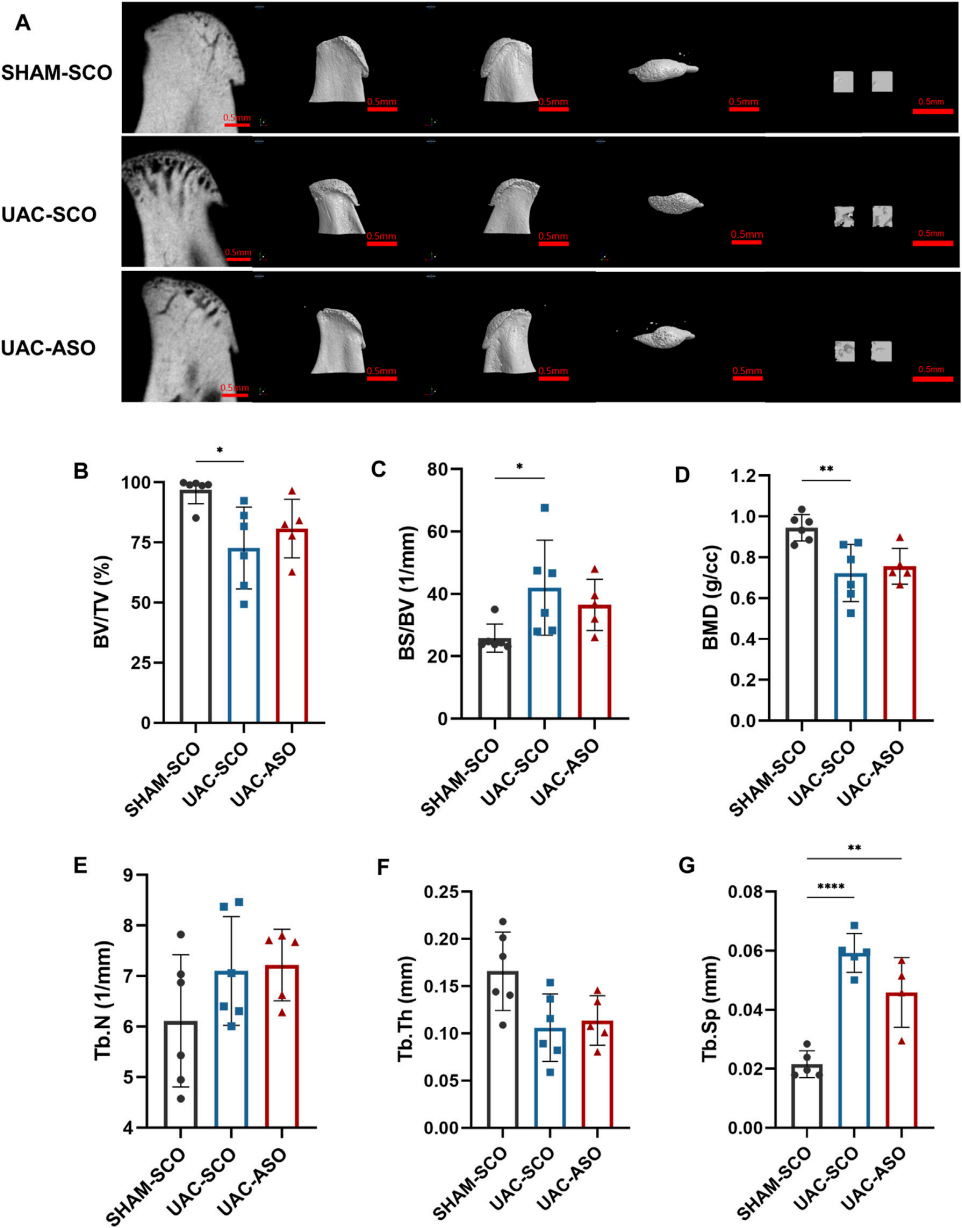
MiR-181a-5p ASO injection restored the condylar Subchondral Bone Quality in TMJOA. **(A)** Representative micro-CT images of condylar head and the selected region of interest (ROI) in SHAM-SCO, UAC-SCO and UAC-ASO mice. For left to right on each row: 2D median sagittal section view, 3D lateral view of left and right side and 3D top-view, 3D reconstruction of the two ROI. For visual comparison, condylar heads in **(A)** are adjusted to the same orientation. Scale bar = 0.5 mm. **(B–G)** Micro-CT data analysis of subchondral bone structure parameters of condyle in the SHAM-SCO, UAC-SCO and UAC-ASO groups. *n* = 6/ group. **(B–G)** Data presented as Scatter dot plot with bar (mean ± SD). ›Statistical analysis was performed using one-way ANOVA with a Tukey’s multiple comparisons test. *p < 0.05; **p < 0.01; ***p < 0.001; ****p < 0.0001.

The UAC-SCO group exhibited obvious subchondral bone destruction compared to the SHAM-SCO group as determined *via* micro-computed tomography, representing a typical TMJOA-like manifestation. Bone destruction was evident in tomography images and 3D reconstruction models (Figure 7A). Reduction of bone mass was the dominant manifestation, and included a reduced percentage of bone volume (BV/TV) (*p* < 0.05), an increased ratio of bone surface to volume (BS/BV) (*p* < 0.05), and significantly reduced bone mineral density (BMD) (*p* < 0.01) (Figures 7B–D). Reduced trabecular bone quality was also identified, as trabecular thickness (Tb.Th) became thinner and there was more trabecular separation (*p* < 0.001) (Tb.Sp) (Figures 7F,G).

No significant subchondral bone recovery was observed after ASO application (Figure 7A). Although reconstructed images derived from UAC-ASO mice suggested that bone surface roughness and trabecular bone damage were lower than they were in the UAC-SCO group, data analysis indicated that BV/TV, BS/BV, and BMD were not significantly improved. Similarly, there were no marked changes in common indicators of trabecular bone (Tb.N, Tb.Th, Tb. Sp) (Figures 7B–G).

## Discussion

An abnormal occlusal relationship is one of the crucial risk factors of TMJ disorder (Xiang et al., 2021). It has been suggested that long-term occlusal disorder leads to TMJ cartilage degeneration, accompanied by upregulation of MMP9 and inflammatory cytokines (Kuang et al., 2013). In the current study unilateral anterior crossbite surgery was performed to simulate malocclusion, which successfully established a stable TMJOA mouse model. In a series of previous studies a sustained abnormal occlusal relationship changed incisal guidance during mastication, resulting in TMJ degenerative and pathological changes, which can mimic TMJOA caused by an abnormal occlusal relationship and excessive joint loading in humans (Li et al., 2020; Liu et al., 2021).

In the present study, after 10 weeks of UAC administration experimental TMJOA exhibited (Liu et al., 2021) thinning of condylar cartilage and a reduced number of chondrocytes; (Shi et al., 2017); degradation of the cartilage matrix; (Li et al., 2021); increased subchondral bone remodeling; and (Wang et al., 2015) and increased rate of chondrocyte apoptosis. These parameters were consistent with previously reported pathological manifestations of early TMJOA (Fang et al., 2021). In prior studies miR-181a-5p was highly expressed in human and animal facet joint and knee osteoarthritis models, suggesting that it may be a crucial mediator involved in articular cartilage degeneration (Nakamura et al., 2019). *In vivo*, miR-181a-5p mimics induced a facet joint osteoarthritis phenotype by initiating cartilage degeneration, chondrocyte loss, proteoglycan reduction, enhanced chondrocyte apoptosis, and increased chondrocatabolism activity (Nakamura et al., 2016; Xue et al., 2018; Nakamura et al., 2019). In the current study, increased miR-181a-5p expression was detected in condylar cartilage of UAC-induced TMJOA mice, indicating that miR-181a-5p also plays an essential role in the pathological TMJOA process. Thus, intervention incorporating miR-181a-5p expression is also expected to alleviate the TMJOA pathological process.

In previous studies, systemic miRNA ASO injection resulted in accumulation in important organs such as the liver and kidney for approximately 2–3 weeks, and also had high stability and affinity after specific modification (Gallo Cantafio et al., 2016). These advantages make miRNA ASO a powerful tool for gene therapy. Systemic administration may induce adverse reactions however, such as neurological injuries (Janssen et al., 2013), cardiomyopathy, and ischemic heart disease (van Zandwijk et al., 2017). Thus, local administration of miRNA ASO into a joint cavity has the advantage of reducing adverse complications, while also targeting a particular tissue. Numerous *in vitro* and *in vivo* experiments of OA exploring ASO-based therapies have provided promising proof in recent years (Nakamura et al., 2020). Kapoor et al. (Nakamura et al., 2019) reported that locked nucleic acid-miR-181a-5p ASO could attenuate cartilage degeneration in facet joint and knee osteoarthritis. Jahr H et al. (Lian et al., 2018) reported that miR-128a-AS facilitated stabilization of chondrocyte autophagy and slowed ACLT-mediated articular tissue destruction of knee joint. Yet, there were no *in vivo* studies exist applicating the miRNA ASO in the TMJOA. In the current study intra-articular injection of miR-181a-5p ASO into the TMJ inhibited miR-181a-5p expression in mice condylar cartilage, playing a protective role and alleviating the degeneration of condylar cartilage in TMJOA.

Protective effects of miR-181a-5p ASO on TMJOA cartilage were observed, but further functional analysis is needed. SIRT1 is a nicotinamide adenine dinucleotide-dependent enzyme silent information regulator 2 type 1 deacetylase that has chondroprotective effects (Deng et al., 2019; Zhou et al., 2021). It is an epigenetic regulator that evidently plays a role in cartilage protection, and the mechanisms involved mainly include regulation of extracellular matrix expression, regulation of bone homeostasis, and anti-catabolic, anti-inflammatory, and anti-apoptotic processes, among others (Deng et al., 2019; Batshon et al., 2020; Zhou et al., 2021). Loss of SIRT1 function can induce apoptosis and aggravate osteoarthritis progression by deacetylating important transcriptional factors and p53, then activating the P53 signaling pathway (Lau et al., 2014; Li et al., 2016; Xu et al., 2020). The SIRT1/P53 signaling pathway is a significant component of chondrocyte hypertrophy, the regulation of apoptosis, and hypersensitivity induced by osteoarthritis (Ong and Ramasamy, 2018). Via database analysis and the dual luciferase reporter assay, the present study demonstrated that miR-181a-5p directly binds to the 3′UTR of SIRT1, and thus negatively regulates SIRT1. Recent studies indicate that SIRT1 is a direct target of miR-181 in epithelial cells, and the negative relationship between them (Sui et al., 2021). SIRT1 activation contributes to the induction of P53 deacetylation, and inhibition of p53-dependent apoptosis (Xu et al., 2020). The current study indicates that miR-181–5p/SIRT1/P53 is a contributory mechanism in TMJOA progression, via regulation of the chondrocyte apoptotic pathway, and that ASO can attenuate this process by inhibiting miR-181–5p. Yan et al. (Yan et al., 2016) have also reported a molecular role of miR-34a in osteoarthritis via regulation of SIRT1/p53 signaling. OA and apoptosis are closely linked (Cazzanelli and Wuertz-Kozak, 2020; Yao et al., 2021). *In vitro* and *in vivo* experimental evidence results of the present study suggested that miR-181a-5p negatively regulates SIRT1 thereby triggering P53 mediated apoptosis in chondrocyte and being involved OA process.

The phenotype of subchondral bone remodeling observed in the present study is notable. Although miR-181a-5p could attenuate cartilage degradation, it did not eliminate pathological changes in subchondral bone. It is likely that the remodeling of cartilage and subchondral bone did not share a regulatory mechanism. Moreover, the relationship between cartilage degradation and subchondral bone destruction under excessive mechanical stress loading remains controversial (Madry et al., 2010; Wei and Dai, 2021). The concept of an “osteochondral unit” is well established, and is potentially informative with respect to the complex relationships between cartilage and subchondral bone, as they may both affect each other (Suri and Walsh, 2012; Goldring and Goldring, 2016). Therefore, we speculated that bone repair may be observed after ASO injection. Notably however, only a protective effect on chondrocytes was evident after miR-181a-5p ASO administration. Generally, more stable joint function protects against subchondral bone loss (Goldring and Goldring, 2016). We considered that the reason for failure of subchondral bone rescue may have been the therapeutic effect of subchondral bone lying behind cartilage, or miR-181a-5p ASO administration missing the therapeutic window of bone repair, because SIRT1 has been identified as a bone formation activator in osteogenic differentiation (Gong et al., 2017). At present, several scholars believe that the destruction or remodeling of subchondral bone precedes cartilage in TMJOA (Zhen and Cao, 2014; Li et al., 2021). This mechanism may explain the inefficiency of cartilage-targeting therapies and hint us that early intervention of subchondral bone may be a promising OA therapeutic strategy (Hu et al., 2021). Additionally, specific time point for treatment also needs careful consideration. There was a time-dependent and extremely complicated evolution of bone remodeling in OA progression (Ni et al., 2022). The weakened supporting force caused by early bone resorption will destroy the osteochondral unit homeostasis, while aberrant mechanical support due to subchondral bone sclerosis will cause further cartilage resorption (Goldring and Goldring, 2016; Hu et al., 2020; Hu et al., 2021). In the current study ASO injection was performed on the 4th week after UAC, when most mice had already developed an obvious TMJOA phenotype in cartilage. Based on this theory and the results of the current study, the optimal therapeutic window for subchondral bone is a point prior to extensive bone destruction. It is of utmost importance that active intervention should be undertaken before the appearance of visible cartilaginous involvement. However, further study is required to identify the exact timepoint for intervention.

## Conclusion

In the current study, increased miR-181a-5p expression participated in the TMJOA process. Intra-articular administration of miR-181a-5p ASO can serve as a gene therapy approach to alleviating the degradation of TMJ cartilage, but not subchondral bone. Mechanically, miR-181a-5p can directly target the 3′UTR site of SIRT1, inducing cellular survival by inhibiting p53-dependent chondrocyte apoptosis.

## Data Availability

The original contributions presented in the study are included in the article/Supplementary Material, further inquiries can be directed to the corresponding author.
