# Supplementary material for: Antisense Oligonucleotide-Based Therapy on miR-181a-5p Alleviates Cartilage Degradation of Temporomandibular Joint Osteoarthritis via Promoting SIRT1
Source: Front Pharmacol. 2022 Jun 15;13:898334. doi: 10.3389/fphar.2022.898334 (PMC9240346; doi:10.3389/fphar.2022.898334)
Supplement: Supplementary file 1 [file Table1.DOCX]

**Antisense oligonucleotide-based therapy on miR-181a-5p** **alleviates cartilage degradation of temporomandibular joint osteoarthritis via promoting SIRT1**

Running title: ASO therapy for TMJOA

Hexu Qi^1#^, Zhenxing Zhao^1,2#^ , Lin Xu^1^, Yue Zhang^3^, Yifei Li^3^, Li Xiao^4^, Yu Li^1^, Zhihe Zhao^1^*, Jie Fang^1^*

Data share link has been listed below, which contains the original/raw microscopic images and mini data sets.

https://www.jianguoyun.com/p/DWaAOZYQ6ZCvChiioLME
